# Cross-Influence between Intra-Laminar Damages and Fibre Bridging at the Skin–Stringer Interface in Stiffened Composite Panels under Compression

**DOI:** 10.3390/ma12111856

**Published:** 2019-06-07

**Authors:** Angela Russo, Andrea Sellitto, Salvatore Saputo, Valerio Acanfora, Aniello Riccio

**Affiliations:** University of Campania “L. Vanvitelli”-Department of Engineering, via Roma, 29, 81031 Aversa, Italy; andrea.sellitto@unicampania.it (A.S.); salvatore.saputo@unicampania.it (S.S.); valerio.acanfora@unicampania.it (V.A.); aniello.riccio@unicampania.it (A.R.)

**Keywords:** debonding, intra-laminar damages, delamination, stiffened panel

## Abstract

In this paper, the skin–stringer separation phenomenon that occurs in stiffened composite panels under compression is numerically studied. Since the mode I fracture toughness and, consequently, the skin–stringer separation can be influenced by the fibre bridging phenomenon at the skin–stringer interface, in this study, comparisons among three different material systems with different fibre bridging sensitivities have been carried out. Indeed, a reference material system has been compared, in terms of toughness performance, against two materials with different degrees of sensitivity to fibre bridging. A robust numerical procedure for the delamination assessment has been used to mimic the skin–stringer separation. When analysing the global compressive behaviour of the stiffened panel, intra-laminar damages have been considered in conjunction with skin–stringer debonding to evaluate the effect of the fibre and matrix breakage on the separation between the skin and the stringer for the three analysed material systems. The latter are characterised by different toughness characteristics and fibre bridging sensitivities, resulting in a different material toughness.

## 1. Introduction

Layered composite materials are usually adopted to produce lightweight structures, able to fulfil specific stiffness and strength requirements, by opportunely stacking a sequence of layers with specific fibre orientations. Therefore, the material performance can be optimised by choosing appropriate fibre orientations according to loading and boundary conditions. However, the usage of composite structures is currently limited by a lack of knowledge of their damage behaviour, especially under compressive loading conditions [1,2,3]. In the case of Carbon-Fibre-Reinforced Polymer (CFRP) laminates, damages are generally triggered by inter-laminar fractures, such as delamination or debonding, that interact with intra-laminar failure mechanisms, such as matrix and fibre breakage. The delaminations in composite material structures have been extensively studied in the literature, both experimentally and numerically. In particular, experimental studies on the composites’ inter-laminar fracture behaviour [4,5,6,7] are mandatory for the development of robust numerical procedures for damage evolution simulation. In [8], three different numerical techniques for the simulation of inter-laminar damage growth in Double-Cantilever Beam (DCB) specimens are proposed. The first two models are based on the Cohesive Zone Model (CZM), while the other model is based on the Virtual Crack Closure Technique (VCCT). Finally, the results have been compared against data from experimental tests. A numerical method, based on the VCCT, and an experimental study are presented also in [9] to examine the fracture behaviour of composite double-cantilever beam specimens. Finite element models for the investigation of the delamination evolution in composites, by means of the VCCT, are described in [10]. Tabiei and Zhang [11] describe and compare the advantages and the drawbacks of the VCCT and the CZM. In [12], the weaknesses in terms of mesh and time-step size dependence of the classical VCCT-based approaches are described. A robust numerical tool for the assessment of inter-laminar damages in composite structures, also based on the VCCT, but able to overcome mesh and time step dependence issues, has been proposed. The real added value of such a numerical procedure, named SMart-time XB (SMXB), is the capability to correctly predict the initiation and the progression of delaminations irrespective of the mesh size and the time increment. Such a tool has been improved in [13] with the capability to mimic the fibre bridging phenomenon by progressively increasing the Mode I fracture toughness *G_Ic_*.

Further studies on delamination onset and propagation can be found in the literature. A numerical and experimental investigation was carried out in [14] to assess the damage behaviour of a woven CFRP laminate subjected to large-deflection bending, considering multiple delaminations. In [15], the interface fracture toughness in curved layered composites is evaluated, and the crack propagation is investigated. An experimental and numerical study on the post-buckling damage behaviour of CFRP-laminated specimens with a cut-out has been presented in [16].

In order to accurately predict the collapse of a composite structure, in-depth knowledge of the interaction between inter-laminar and intra-laminar failure mechanisms is mandatory. Indeed, matrix and fibre cracks affect delamination initiation and evolution [17,18]. Moreover, intra-laminar cracks operate as delamination migration paths among adjacent plies or as boundaries that limit delamination evolution [19,20]. In [21], the SMXB procedure has been coupled to a User Material Subroutine [22] for the modelling of intra-laminar damages by means of instantaneous degradation of the material properties based on the Hashin’s failure criteria. A single stiffened CFRP panel was studied, and comparisons were made between numerical results and experimental data.

Following the study presented in [21], the cross-influence between intra-laminar damages and delamination initiation and growth has been assessed in this work. The procedure developed in [13] takes into account the effect of the fibre bridging on the delamination growth and has been used in conjunction with Hashin’s failure criteria [23,24] to numerically reproduce the intra-laminar damages. Moreover, the compressive behaviour of the delaminated I-stiffened composite panel has been numerically analysed. The interaction between the damage mechanisms has been analysed for different material systems, and were obtained by opportunely tuning the manufacturing process. The analysed material systems are characterised by different toughness characteristics and fibre bridging sensitivities, involving a different Mode I fracture toughness. The SMXB procedure, in conjunction with the Hashin’s criteria and material property degradation rules, has been used. The effects of fibre bridging and fibre/matrix breakage on delamination growth have been considered by opportunely varying *G_Ic_* when the delamination front advances. In Section 2, the theoretical background of the SMXB procedure and Hashin’s intra-laminar failure criteria, with the material property degradation rules, are described. In Section 3, the finite element modelling activity is introduced. Finally, comparisons between the numerical results, obtained by taking into account only inter-laminar damage and both intra-laminar and inter-laminar damages, for different fibre bridging sensitivities are discussed in Section 4.

## 2. VCCT-Based Numerical Procedure (SMXB) 

In the following paragraphs, the theory behind the numerical models adopted in this work is described. The SMXB procedure, introduced in [13], for the simulation of the inter-laminar damage’s evolution is briefly explained. It underlies the capability to evaluate crack propagation irrespective of the mesh and time-step size and to take into account the fibre bridging phenomenon. Finally, the adopted Hashin’s criteria are introduced.

### 2.1. SMXB Methodology

Using the Ansys Parametric Design Languages (APDL), a new numerical methodology has been implemented in the Ansys^®^ Finite Element Model (FEM) simulation software. SMXB is described in detail in [12,13]. It is the result of an improved combination of the Virtual Crack Closure Technique (VCCT) and the Fail release (FR) method. In particular, the proposed methodology is able to overcome the main limitations of the standard VCCT-FR approaches [12] in terms of dependence on mesh and load-step size. The potential area of interest with the delamination growth is simulated by means of contact elements and fail-release conditions, which can be deactivated where the propagation criterion is fulfilled. The criterion used in SMXB is the Linear Power Law, which is described by Equation (1).

(1) $$E_{d}=\frac{G_{I}}{G_{Ic}}+\frac{G_{II}}{G_{IIc}}+\frac{G_{III}}{G_{IIIc}}\geq 1$$

The term *G_j_* (with *j = I, II, III*) is the energy release rate, related to the failure mode *j*, while *G_jc_* is the corresponding critical value.

**Figure 1:**
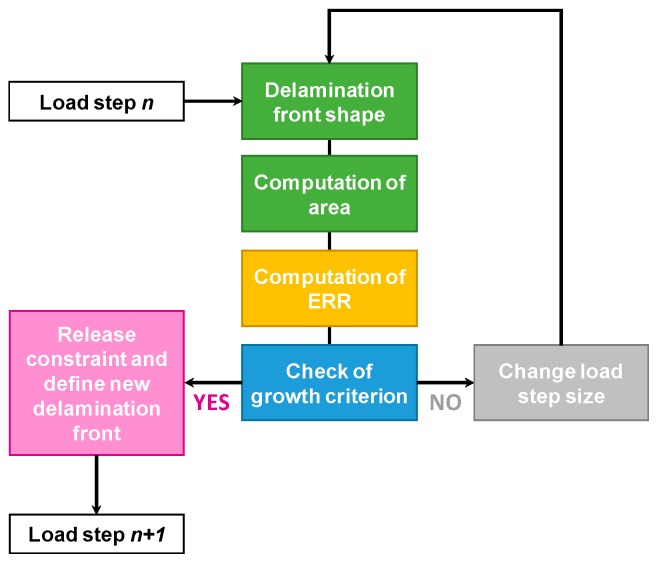
Smart-time XB (SMXB) flow chart.

The delamination growth simulation strongly depends on the size of the elements. The delaminated area can generally be under- or overestimated. Through the combination of three separate and interacting moduli, the SMXB technique is able to change the local coordinate system related to each node, belonging to the delamination front, to properly determine the virtually released area. This allows it to equate the numerically evaluated delaminated area with the area that should be released to reach *E_d_* = 1. Furthermore, the peaks in the strain energy release rate that occur at corner nodes, due to the change in the delamination front’s shape, are avoided by estimating the energy release rate on the delamination front’s segments, rather than nodes. In Figure 1, a flowchart representing the SMXB workflow is shown. The detailed description of SMXB features is reported in [12,13].

### 2.2. Fibre Bridging Module

**Figure 2:**
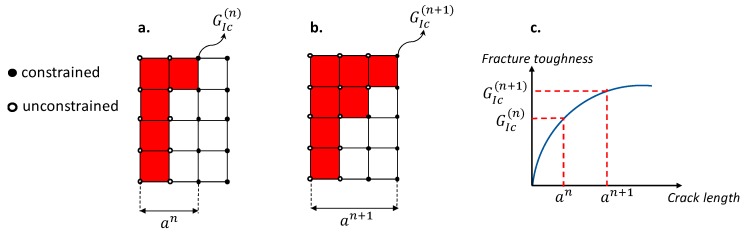
Fibre bridging modulus.

During delamination evolution, fibre-reinforced composite materials can show crack bridging, such as fibres crossing the open crack. Such a phenomenon, namely fibre bridging, increases the fracture toughness of the material as the crack length grows (see Figure 2) and, consequently, it is able to delay the delamination evolution. In [13], the SMXB procedure has been improved by implementing a modulus that is able to mimic the fibre bridging phenomenon by considering the *G_Ic_* variations induced by fibre bridging (R-Curve, Figure 2c). Essentially, the *G_Ic_* value changes as the constraints between nodes, representing the delaminated area, are released, as shown in Figure 2. Therefore, the fracture toughness grows according to the R-curve until a steady state value, which is representative of the maximum extension of the fibre bridging, is reached.

According to the theory underlying the fibre bridging modulus, the formula in Equation (1) should be written (where *a* is the crack extension) as:

(2) $$E_{d}=\frac{G_{I}}{G_{Ic}\left(a\right)}+\frac{G_{II}}{G_{IIc}}+\frac{G_{III}}{G_{IIIc}}\geq 1.$$

### 2.3. Hashin Criteria and Material Property Degradation Rules

Hashin and Rotem in [23,24], on the basis of experimental evidence, propose for unidirectional composite materials the presence of two different intra-laminar mechanisms of failure that are dominated by the fibre phase and the matrix phase. A criterion for each of these two distinct failure modes has been introduced. In particular, under compressive load in the fibres’ direction, a simple criterion of maximum stress is proposed, as shown in Equation (3).

(3) $${\left(\frac{\sigma_{1}}{X_{c}}\right)}^{2}=1\;\mathrm{with}\;\sigma_{1}<0$$

In the case of tensile fibre failure, the expression in Equation (4) can be used.

(4) $${\left(\frac{\sigma_{1}}{X_{t}}\right)}^{2}+{\left(\frac{\sigma_{12}}{S}\right)}^{2}=1\;\mathrm{with}\;\sigma_{1}\geq 0$$

In the case of transverse stress, the expression for predicting matrix failure under tensile stress is described by Equation (5), and, if the stress is compressive, Equation (6) should be considered.

(5) $${\left(\frac{\sigma_{2}}{Y_{t}}\right)}^{2}+{\left(\frac{\sigma_{12}}{S}\right)}^{2}=1\;\mathrm{with}\;\sigma_{2}\geq 0$$

(6) $${\left(\frac{\sigma_{2}}{2S}\right)}^{2}+{\left(\frac{\sigma_{12}}{S}\right)}^{2}+\left[{\left(\frac{Y_{c}}{2S}\right)}^{2}-1\right]\cdot \frac{\sigma_{2}}{Y_{c}}=1\;\mathrm{with}\;\sigma_{2}<0$$

According to Equations (3)–(6), *σ_1_* and *σ_2_* are the stresses in the fibre direction and normal to the fibre direction, respectively; and *X_c_*, *Y_c_*, *X_t_*, *Y_t_* and *S* are the fibre compressive, matrix compressive, fibre tensile, matrix tensile, and shear strengths, respectively.

**Figure 3:**
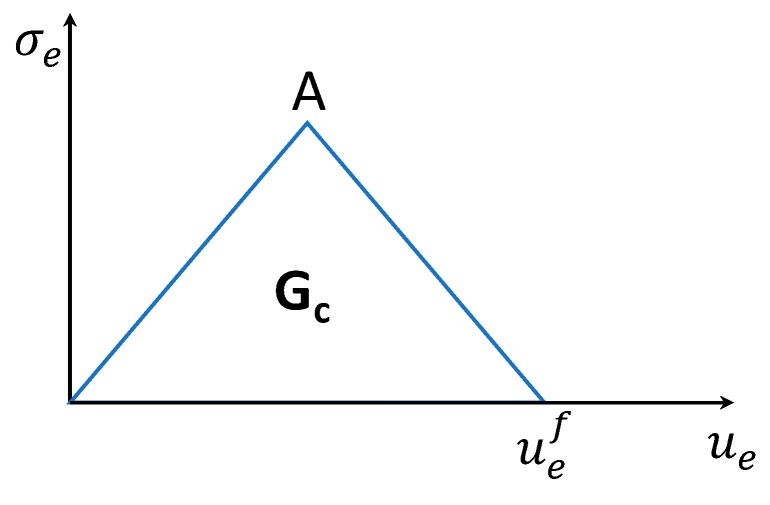
Linear material softening.

The Damage Evolution Law, which defines the way the material degrades, is shown in Figure 3.

Location A in Figure 3 represents Hashin’s stress limit. When this location is reached, the material stiffness is reduced by means of degradation factor *d*. Following the onset of damage, a reduction in material stiffness occurs immediately. The constitutive relationship for a damaged material is given by Equation (7), where *σ* is the nominal Cauchy stress, *ε* is the total elastic strain and [*D*]*_d_* is the damaged elasticity matrix.

(7) $$\sigma ={\left[D\right]}_{d}\varepsilon$$

For a transversely isotropic material in a plane stress state, the damaged elasticity matrix can be expressed as in Equation (8),

(8) $$C=\frac{1}{D}\left[\begin{array}{ccc} (1-d_{f})E_{1} & (1-d_{f})\left(1-d_{m}\right)\nu_{21}E_{1} & 0\\ \left(1-d_{f}\right)\left(1-d_{m}\right)\nu_{12}E_{2} & \left(1-d_{m}\right)E_{2} & 0\\ 0 & 0 & D\left(1-d_{s}\right)G \end{array}\right]$$

where $D=1-\left(1-d_{f}\right)\left(1-d_{m}\right)\nu_{12}\nu_{21}>0$. The terms *d_s_*, *d_m_* and *d_f_* represent the shear, matrix and fibre damage variables, respectively, that can be calculated as in Equation (9),

(9) $$\begin{array}{l} \left\{ \begin{array}{l} d_{f}^{+}\;\mathrm{if}\;\lambda_{f}^{+}>0\\ d_{f}^{-}\;\mathrm{if}\;\lambda_{f}^{-}>0 \end{array}\right.\\ \left\{ \begin{array}{l} d_{m}^{+}\;\mathrm{if}\;\lambda_{m}^{+}>0\\ d_{m}^{-}\;\mathrm{if}\;\lambda_{m}^{-}>0 \end{array}\right.\\ d_{s}=1-\left(1-d_{t}{}^{+}\right)\left(1-d_{f}{}^{-}\right)\left(1-d_{m}{}^{+}\right)\left(1-d_{m}{}^{-}\right) \end{array}$$

where *λ_f_*^+^, *λ_f_*^−^, *λ_m_*^+^ and *λ_m_*^−^ are the fibre tension, fibre compression, matrix tension and matrix compression failure, respectively, evaluated from the effective stress *σ*. Two damage evolution approaches are available: instantaneous degradation and gradual degradation. The first approach, which has been considered in this work, instantaneously reduces the material stiffness based on damage variables. According to the second approach, the damage variables progressively grow.

## 3. Numerical Model

**Figure 4:**
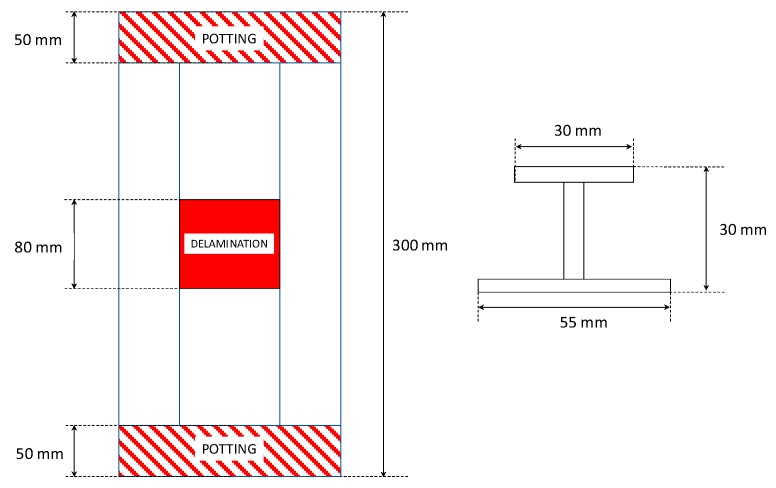
Geometrical model.

**Figure 5:**
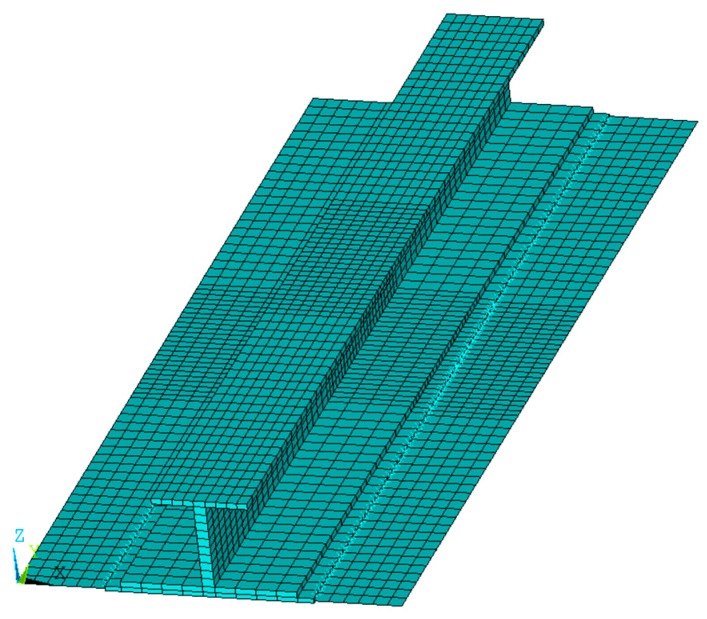
Finite element model (FEM).

An I-stiffened reinforced composite panel, with induced delamination between the panel and the stringer, has been considered in this work. The geometrical description is shown in Figure 4. Twenty solid layered node elements have been considered to discretize the reinforcement and the skin underneath, while linear layered structural shell elements have been used to mesh the remaining skin panel, as shown in Figure 5, in order to reduce the computational cost [25]. The model consists of 2730 solid elements and 840 shell elements. The overall number of nodes is 22,837.

In the framework of the performed numerical analyses, the panels’ borders have been bounded, taking into account the effect of the potting regions in order to assure an even distribution of the load. The panel and the reinforcement have been linked, in the area where delamination is expected to grow, by using contact elements with “birth and death” features. Furthermore, node-to-surface contact elements have been positioned in the delaminated area to prevent penetration. Compressive displacements have been imposed on one side of the panel, and the other side has been clamped. Non-linear analyses have been performed, with variable time-steps, according to the SMXB procedure. Indeed, the numerical tool iteratively changes the time-step’s size until *E_d_* = 1 is reached. Furthermore, no warping phenomena are expected to occur due to the symmetrical and balanced stacking sequence and the boundary conditions.

The mechanical properties of the used fibre-reinforced composite materials are listed in Table 1. Three different material models, with different *G_Ic_* values, have been considered:

- a reference material with *G_Ic_* = 0.243 kJ/m^2^, which was obtained by means of the standard Solvay curing process;
- a toughened material with *G_Ic_* = 0.456 kJ/m^2^, which was obtained by means of a modified curing process aimed at improving the fracture toughness; and
- a high fibre bridging material, with an increasing value of *G_Ic_*, which was obtained by means of a modified curing process and characterised by high sensitivity to the fibre bridging phenomenon.

**Figure 6:**
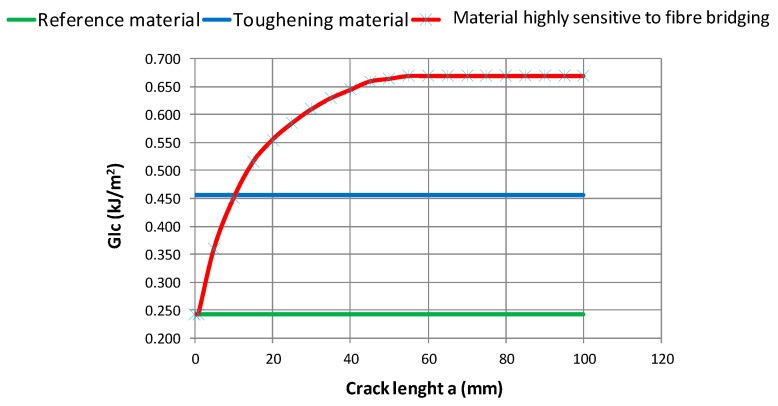
*G_Ic_* vs. Crack length *a*.

The resistance curve, characterising the material as highly sensitive to the fibre bridging, is shown in the chart in Figure 6, and is compared with the constant values of *G_Ic_*.

## 4. Results

In this section, the results of the proposed numerical analyses are presented and discussed for all the considered material systems. The adopted numerical approach, introduced in Section 3, has been extensively investigated and validated by means of comparisons with experimental test results. These studies can be found in [13,21]; however, they are not reported in this work for the sake of brevity.

The compressive behaviour of the I-stringer panels, considering only the inter-laminar damages, is initially presented. Afterward, the Hashin’s criteria have been added to the numerical simulations, and the effect of the fibre/matrix breakage on the debonding evolution has been studied.

### 4.1. Debonding Simulations: Material Sensitivity Analysis

**Figure 7:**
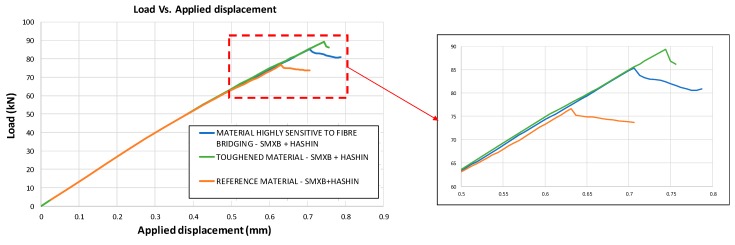
Load vs. Applied displacement (SMXB).

The global compressive behaviour of the analysed configurations, considering only the inter-laminar damage evolution, is reported in Figure 7, where the load versus applied displacement curves are compared for the three different analysed materials. It has been found that the delamination onset is influenced by the material’s characteristics. Indeed, in the reference material model, the maximum attained load is 76.6 kN as compared to the 85.4 kN of the material that is highly sensitive to the fibre bridging phenomenon. On the other hand, the toughened material model reaches a maximum load of 90 kN.

**Figure 8:**
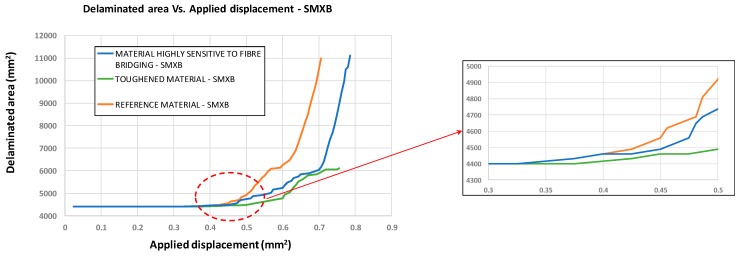
Delaminated area vs. Applied displacement (SMXB).

In Figure 8, the results in terms of delaminated area versus applied displacements are introduced for the three analysed materials. Even if the fibre bridging phenomenon has been shown to delay the debonding evolution, the trend of the delaminated area does not change when considering the reference material and the material sensitive to the fibre bridging. Indeed, both are characterised by a stable separation evolution until a sudden collapse occurs, which corresponds to a complete loss of stiffness (maximum load in Figure 7). Debonding arises simultaneously in the reference material model and the configuration sensitive to the fibre bridging. In the toughened material configuration, skin–stringer separation onset is delayed and the debonding growth is more stable (as can be seen from the reduced curve slope) and seems to reach a steady state. This is because the starting value of *G_Ic_* is the same for the configurations that are characterised by a reference material system and a material system with high sensitivity to fibre bridging (0.243 kJ/m^2^ according to the graph in the Figure 6), while the value is higher for the configuration characterised by the toughened material systems (the blue curve in the Figure 6), where there is a delay in delamination onset. Overall, it can be observed that the Mode I fracture toughness has a significant influence on the panel-reinforcement separation’s progression. In particular, increasing the Mode I fracture toughness value stabilises the inter-laminar damage progression.

**Figure 9:**
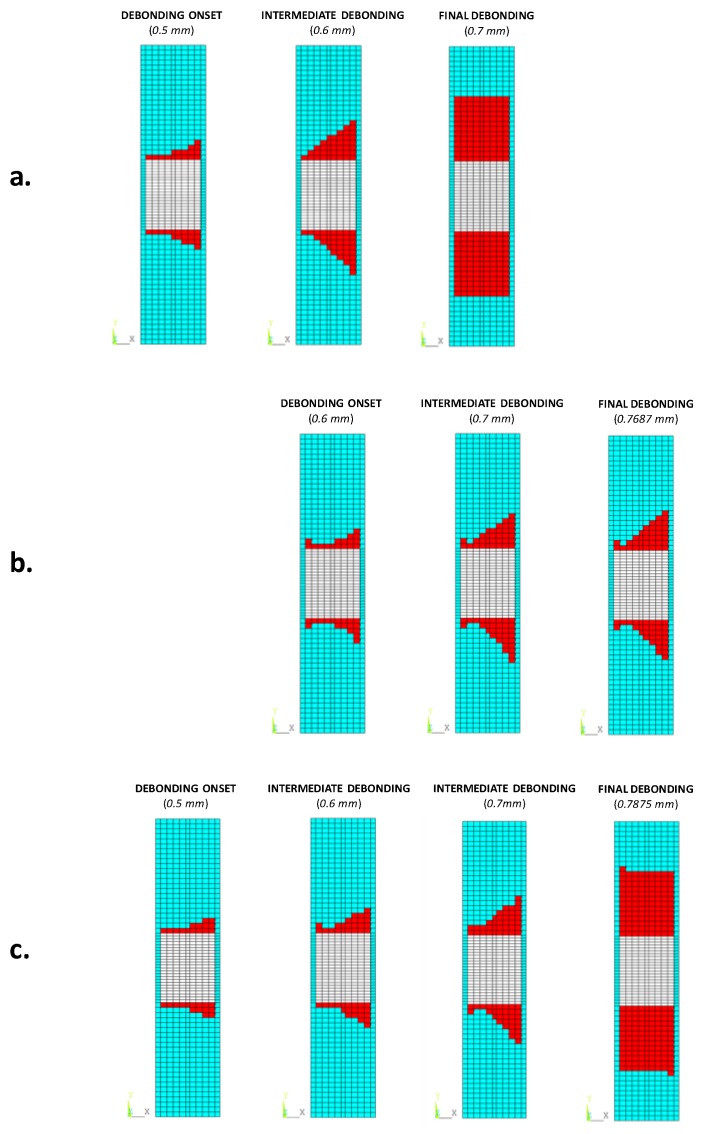
Delamination evolution in: (**a**) the reference material configuration; (**b**) the toughened material configuration; (**c**) the material configuration with high sensitivity to fibre bridging (SMXB).

The debonding evolution trends, at different applied compressive displacement values, are shown in Figure 9 for each analysed configuration. According to the figure, the debonding fronts grow with an inclination of about 45° with respect to the loading direction.

**Figure 10:**
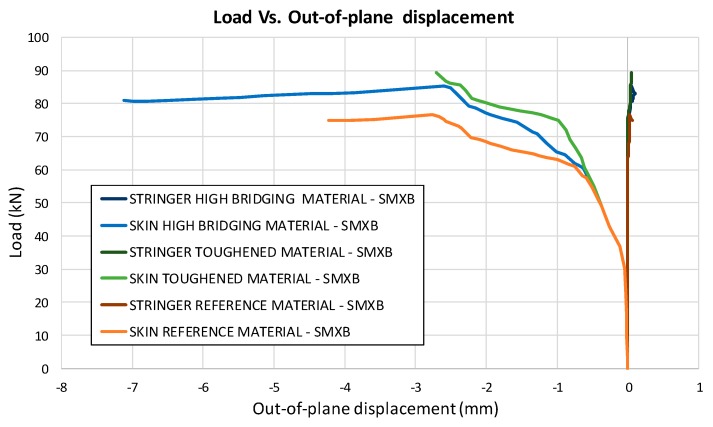
Load vs. Out-of-plane displacements (SMXB).

In Figure 10, the load versus the out-of-plane displacement curves, representative of the buckling behaviour of the panels, are shown for each analysed configuration. Local buckling of the delaminated region can be appreciated at 30 kN. The delamination growth initiation load values, together with the buckling loads, are listed for each analysed configuration in Table 2. The delamination growth initiation load for the reference material is the same as that for the material with high sensitivity to fibre bridging. Indeed, for these two materials, the value of the critical energy release rate at the beginning of delamination growth is the same.

### 4.2. Cross-Influence between Intra-Laminar Damages and Fibre Bridging

**Figure 11:**
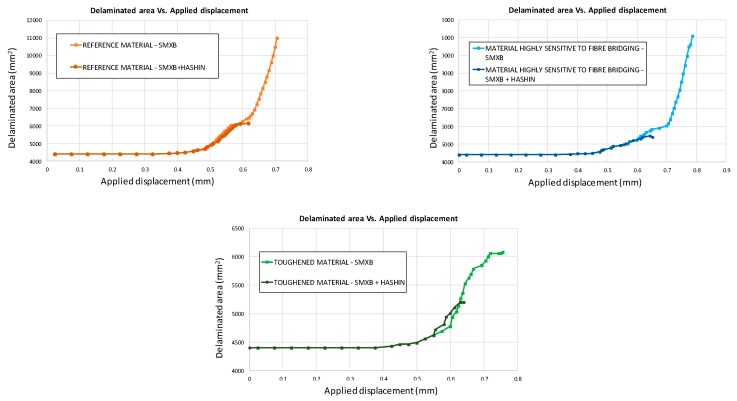
Delaminated area vs. Applied displacement comparison.

The three different panel configurations (characterised by different material systems) have been further analysed by considering both the inter-laminar and the intra-laminar damage evolution. The influence of the fibre/matrix breakage on the delamination evolution can be observed in Figure 11, where the delaminated areas found with the SMXB procedure are compared with the delaminated areas found by using SMXB coupled with the Hashin’s criteria, for all the considered material models.

**Figure 12:**
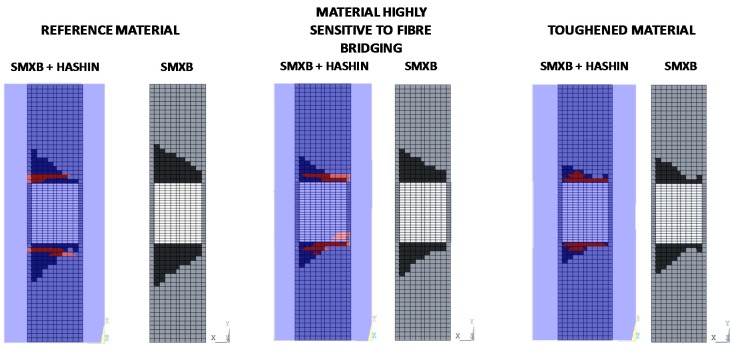
Delamination evolution comparison.

**Figure 13:**
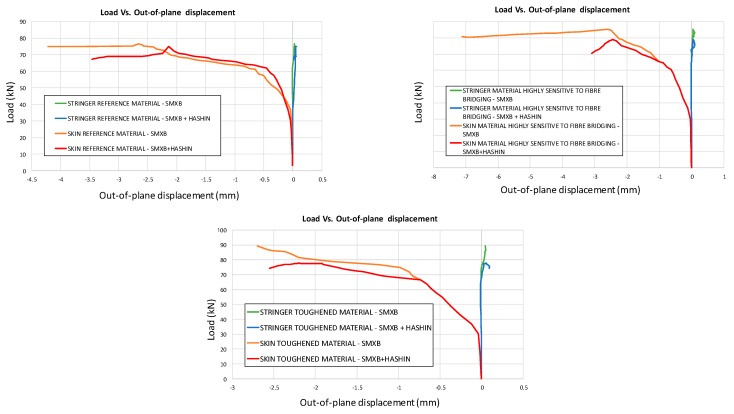
Load vs. Out-of-plane displacement comparison.

**Figure 14:**
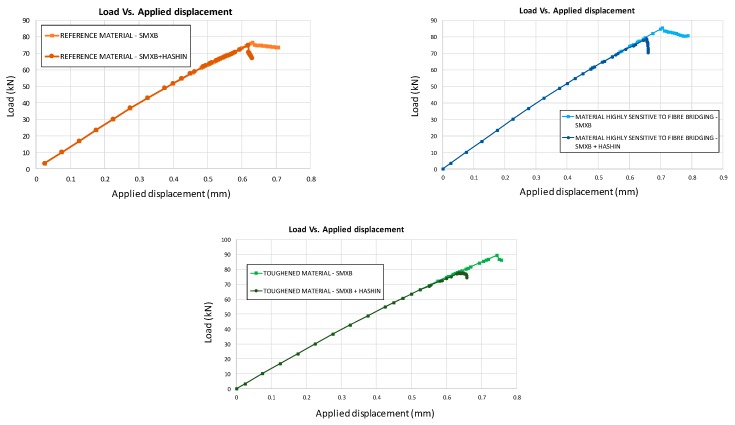
Load vs. Applied displacement comparison.

According to the charts in Figure 11, the debonding onset and evolution are the same as in the previously presented analyses up to the fibre/matrix damage onset. Indeed, the fibre’s breakage arises along the delamination front, influencing the delamination evolution, as shown in Figure 12. The debonded areas that were found with the SMXB procedure are compared with the debonded areas that were found by using SMXB coupled with the Hashin criteria, at the same applied displacement value, which corresponds to the final state of each analysed material system found by using the SMXB approach coupled with the Hashin’s criteria. In Figure 12, the fibre’s failure is superimposed in red on the images reporting the delamination evolution status. According to Figure 12, it can be observed that the intra-laminar damages arising on the delamination front influence the delamination evolution, resulting in a reduction of the delaminated area. However, despite the reduction in inter-laminar damage, the intra-laminar damages critically reduce the structure’s load carrying capability, as shown in Table 3 and Figure 13 and Figure 14. In particular, in Table 3, the bucking load and applied displacement values, together with the intra-laminar and inter-laminar onset load and applied displacement values, are listed, for each analysed configuration. Figure 13 shows the load versus the out-of-plane displacement comparisons. According to Table 3 and Figure 13, the onset of local buckling in the delaminated region, which takes place before the onset of intra-laminar damage, is not affected by the intra-laminar damage. On the contrary, the appearance of the intra-laminar damages modifies the global compressive behaviour of the panels, leading prematurely to failure before the complete development of the buckling phenomenon seen in the previously shown SMXB analyses.

Moreover, the global compressive behaviour, considering the coupled intra-laminar and inter-laminar damages, seems to be different both in the evolution of delaminated areas and in load trends, which are shown in Figure 14. As expected, the maximum load decreases when introducing the intra-laminar damage, which anticipates a structural collapse with respect to the ideal case analysed with the SMXB model.

**Figure 15:**
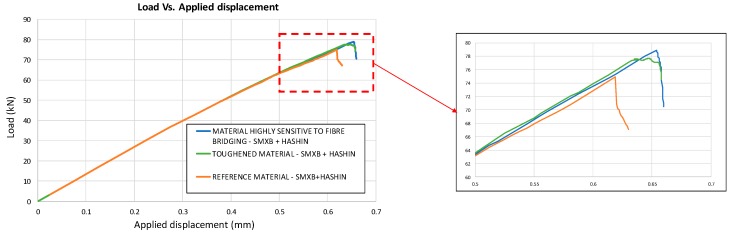
Load vs. Applied displacement (SMXB+HASHIN).

In Figure 15, the load versus displacement curves, related to the three analysed material systems with combined intra-laminar and inter-laminar damage evolution, are shown. From this figure, it is possible to appreciate that the Mode I fracture toughness values do not have a significant influence on the global behaviour of the analysed configurations under compression. However, the maximum attained load in the case of the reference material system remains lower than that of the other configurations, which have a higher Mode I fracture toughness.

**Figure 16:**
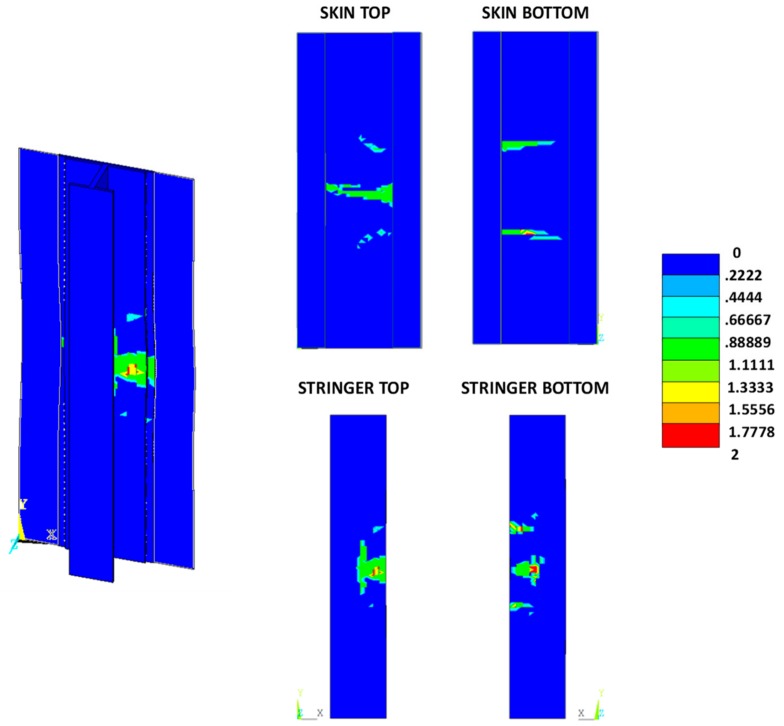
Damage status: reference material system.

**Figure 17:**
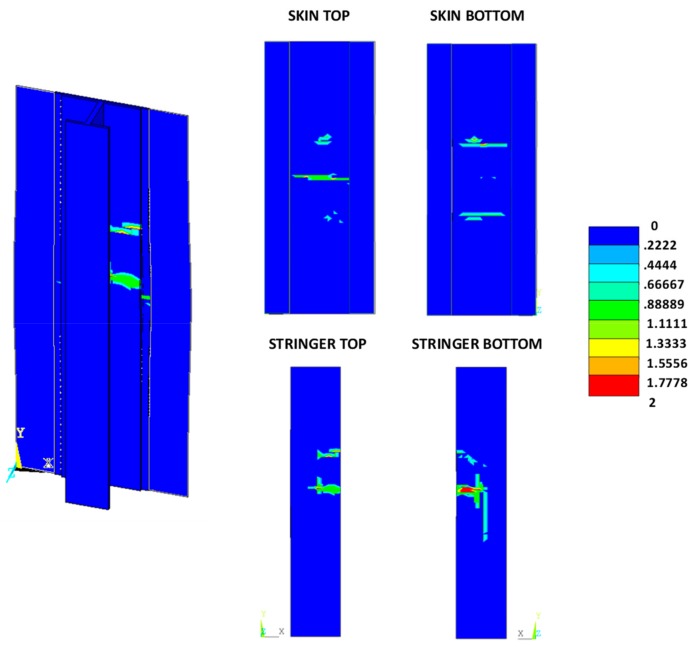
Damage status: toughened material system.

**Figure 18:**
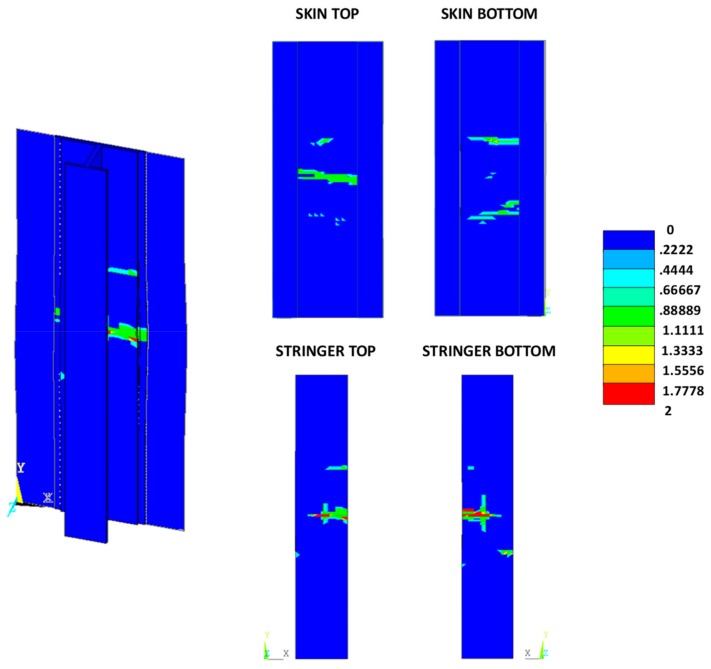
Damage status: material system with high sensitivity to fibre bridging.

The intra-laminar damage status, for the three analysed configurations, at the final states, are shown in Figure 16, Figure 17 and Figure 18. Contour values over unity indicate a damaged region, while zero indicates an undamaged region (0, undamaged; 1, damaged; 2, completely damaged).

From Figure 16, Figure 17 and Figure 18, as expected, no relevant differences between the three analysed material systems, in terms of intra-laminar damage distribution, can be noted. The intra-laminar damage caused a loss in the carrying capability of the panel, regardless of the inter-laminar damage onset load.

**Figure 19:**
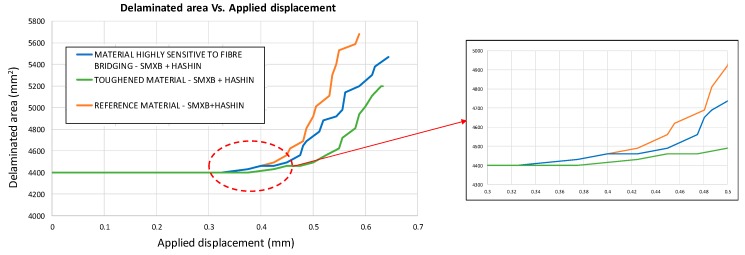
Delaminated area vs. Applied displacement (SMXB+HASHIN).

Figure 19 shows the delaminated area versus the applied displacements for the three considered configurations. It is clear from the chart that an increase in Mode I toughness reduces the delaminated area even in the presence of intra-laminar damage. Indeed, as an example, considering the fixed applied displacement value of 0.55 mm, the delaminated area can be reduced by up to 16.5% by increasing the Mode I fracture toughness.

## 5. Conclusions

In this paper, the influence of intra-laminar damages on the evolution of skin–stringer debonding in stiffened composite panels under compression has been investigated. Three different material systems have been analysed, taking into account the fibre bridging phenomenon. A validated robust numerical procedure, (SMXB), has been used for the numerical analyses. The proposed methodology is able to simulate the delamination evolution, in the presence of fibre bridging, with no mesh and time-step size dependency. First, a set of numerical simulations was carried out in which only inter-laminar damages were considered. It was found that, by increasing the Mode I fracture toughness, a significant decrease in delamination evolution can be obtained. In particular, the material systems with increased fracture toughness experience stable debonding growth and seem to reach a steady state condition. A second set of analyses was carried out by taking into account the evolution of intra-laminar damage in order to assess the influence of fibre/matrix breakage on the delamination evolution. The results show that the maximum achieved load is reduced and that the intra-laminar damages take place at the delamination front, influencing the delamination evolution and resulting in a reduction of the delaminated area. In conclusion, it was also observed that the value of the fracture toughness of the interface does not influence the fibre/matrix failure growth.

## Figures and Tables

**Table 1:** Material properties.

Parameter	Value
E_11_	147,000 (MPa)
E_12_	8500 (MPa)
G_12_ = G_13_	4500 (MPa)
G_23_	4000 [MPa)
ν_12_ = ν_13_	0.36 (-)
ν_23_	0.45 (-)
G_IIc_ = G_IIIc_	0.514 (kJ/m^2^)
X_T_	1022 (MPa)
X_C_	614 (MPa)
Y_T_	54 (MPa)
Y_C_	169 (MPa)
S_L_	63 (MPa)
S_T_	28 (MPa)
Stacking sequence skin	(0, 90, +45, −45)s
Stacking sequence top/foot stringer	(0, 0, +45, −45, 0)s
Stacking sequence web stringer	(0, 0, +45, −45, 0)2s
Ply thickness	0.190 mm

**Table 2:** Delamination onset and buckling load (SMXB).

	Buckling		Delamination Growth Initiation	
	Applied Displacement (mm)	Load (kN)	Applied Displacement (mm)	Load (kN)
Reference material	0.325	42.9	0.375	48.9
Toughened material	0.325	42.9	0.425	54.9
Material with high sensitivity to fibre bridging	0.325	42.9	0.375	48.9

**Table 3:** Delamination onset, buckling, and fibre breakage load (SMXB+HASHIN).

	Buckling		Delamination		Fibre Damage	
	Applied Displacement (mm)	Load (kN)	Applied Displacement (mm)	Load (kN)	Applied Displacement (mm)	Load (kN)
Reference material	0.325	42.9	0.375	48.9	0.531	66.4
Toughened material	0.325	42.9	0.425	54.9	0.525	66.5
Material with high sensitivity to fibre bridging	0.325	42.9	0.375	48.9	0.519	65.3
